# TFEB Orchestrates Stress Recovery and Paves the Way for Senescence Induction in Human Dermal Fibroblasts

**DOI:** 10.1111/acel.70083

**Published:** 2025-05-01

**Authors:** Lena Guerrero‐Navarro, Pablo Monfort‐Lanzas, Vinzenz Krichbaumer, Mariana E. G. De Araújo, Jlenia Monfregola, Lukas A. Huber, Andrea Ballabio, Pidder Jansen‐Dürr, Maria Cavinato

**Affiliations:** ^1^ Institute for Biomedical Aging Research Universität Innsbruck Innsbruck Austria; ^2^ Center for Molecular Biosciences Innsbruck (CMBI) Innsbruck Austria; ^3^ Institute of Medical Biochemistry Biocenter, Innsbruck Medical University Innsbruck Austria; ^4^ Institute of Bioinformatics Biocenter, Innsbruck Medical University Innsbruck Austria; ^5^ Biocenter, Division of Cell Biology Innsbruck Medical University Innsbruck Austria; ^6^ Telethon Institute of Genetics and Medicine (TIGEM) Naples Italy

**Keywords:** mTOR, senescence, SIPS, tBHP, TFEB

## Abstract

Cells experience oxidative stress and widespread cellular damage during stress‐induced premature senescence (SIPS). Senescent cells show an increase in lysosomal content, which may contribute to mitigating cellular damage by promoting autophagy. This study investigates the dynamics of lysosomal quality control in human dermal fibroblasts (HDF), specifically examining lysosomal signaling pathways during oxidative stress‐induced SIPS. Our results reveal distinct signaling responses between the initial stress phase and the ensuing senescent phenotype. During the stress phase, treatment with tBHP, which undermines the antioxidant response, leads to elevated reactive oxygen species (ROS) and lysosomal damage. ROS accumulation activates AMP‐activated protein kinase (AMPK) and inhibits Akt, which correlates with the suppression of mammalian target of rapamycin (mTOR). Inactivation of mTOR during this phase aligns with the activation of transcription factor EB (TFEB), a key regulator of autophagy and lysosomal biogenesis. TFEB knockdown under stress increased apoptosis, highlighting the protective role of TFEB in the stress response. As cells transition to senescence, TFEB activity, required for the autophagic damage repair, becomes less critical. The decrease in ROS levels leads to the normalization of AMPK and Akt signaling, accompanied by the reactivation of mTOR. This reactivation of mTOR, which is critical for establishing the senescent state, is observed alongside the inactivation of TFEB. Consequently, as damage decreases, TFEB activity decreases. Our results suggest a dynamic interplay between TFEB and mTOR, highlighting a critical role of TFEB in ensuring cellular survival during SIPS induction but becoming dispensable once senescence is established.

## Graphical abstract

TFEB dynamics during oxidative stress‐induced premature senescence. The tBHP treatment undermines GSH‐dependent antioxidant mechanisms, leading to increased ROS production and lysosomal damage. In the stress phase, we observed the concomitant inhibition of Akt along with activation of AMPK, together leading to mTOR inhibition and subsequent dephosphorylation of TFEB which is known to drive its nuclear localization and activation. Inside the nucleus, TFEB activates the transcription of the Coordinated Lysosomal Expression and Regulation (CLEAR) network genes, enhancing lysosomal biogenesis, and antioxidant capacity, thereby promoting lysosomal recovery, autophagy and subsequent entry into the senescent state. Decreased ROS levels restore Akt and AMPK pathways, leading to the resurgence of mTORC1 activity, along with TFEB inactivation as the cells transit into senescence.

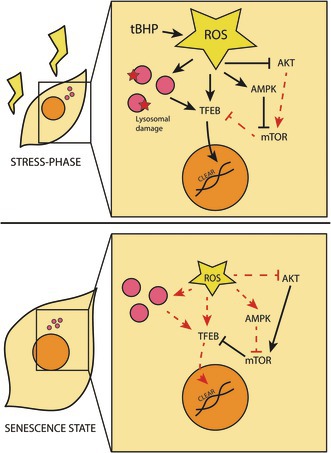

**Abbreviations**
AMPK: AMP‐Activated Protein Kinase
CHMP4B: Charged Multivesicular Body Protein 4B
DDR: DNA Damage Response
ESCRT: Endosomal Sorting Complex Required for Transport
HDF: Human Dermal Fibroblasts
LAMP1: Lysosomal Associated Membrane Protein 1
LC3: Microtubule‐Associated Proteins 1A/1B Light Chain 3B
mTOR: Mammalian Target of Rapamycin
OIS: Oncogene‐Induced Senescence
ROS: Reactive Oxygen Species
SASP: Senescence‐Associated Secretory Phenotype
SA‐β‐gal: Senescence‐Associated Beta‐Galactosidase
SIPS: Stress‐Induced Premature Senescence
tBHP: tert‐Butyl Hydroperoxide
TFEB: Transcription Factor EB

## 1 Introduction

Aging is a complex biological process characterized by a gradual decline in physiological functions, leading to an increased vulnerability to age‐related diseases. In many tissues, the aging process correlates with the accumulation of senescent cells (López‐Otín et al. 2023), which cease to divide but remain metabolically active. A hallmark feature of senescent cells is the persistent activation of the DNA damage response (DDR). The persistent DDR leads to the upregulation of pivotal tumor suppressor pathways, notably the p53/p21 and p16/pRb pathways, both of which are instrumental in enforcing cell cycle arrest (Kohli et al. 2021). Senescent cells secrete a variety of factors known collectively as the Senescence‐Associated Secretory Phenotype (SASP), which is thought to represent the main mediator of the pathological effects that senescent cells cause in the context of aging (López‐Otín et al. 2023).

While cellular senescence shares fundamental characteristics in different cell types, such as cell cycle arrest and a persistent DDR, it is inherently heterogeneous. The specific characteristics of senescent cells, including the composition of the SASP, vary depending on the cell type and the stimulus that induced senescence. Different triggers initiate senescence, including telomere shortening, which leads to replicative senescence (Campisi 1997), and oncogene activation, resulting in oncogene‐induced senescence (OIS) (Courtois‐Cox et al. 2008). Additionally, exposure to damaging agents such as ionizing radiation, ultraviolet light, H_2_O_2_, or other stressors can result in stress‐induced premature senescence (SIPS) (de Magalhães and Passos 2018). SIPS is marked by an increase in oxidative stress and extensive cellular damage. To counteract the increase in reactive oxygen species (ROS) production and maintain viability, cells activate protective pathways (Espinosa‐Diez et al. 2015).

Macroautophagy (hereafter referred to as autophagy) is a fundamental cellular process crucial for maintaining cellular homeostasis and integrity, especially under conditions of stress (Kroemer et al. 2010). Autophagy is a self‐degradative mechanism that enables cells to recycle cytoplasmic components and dispose of dysfunctional organelles and proteins. During autophagy, cellular components are sequestered within double‐membraned vesicles called autophagosomes, which then fuse with lysosomes, where the contents are degraded and recycled (Kroemer et al. 2010).

Thus, lysosomes play a crucial role in the autophagic degradation and recycling of macromolecules and damaged organelles, thereby supporting cellular metabolism and energy production (Lawrence and Zoncu 2019). Due to their role, lysosomal quality control mechanisms are critical for maintaining cellular function and homeostasis. These mechanisms include repair through the Endosomal Sorting Complex Required for Transport (ESCRT) machinery, which seals lysosomal membrane breaches (Yang and Tan 2023), and lysophagy, a selective autophagic process that targets damaged lysosomes for degradation (Yang and Tan 2023). Beyond these strategies, lysosomal biogenesis is a pivotal response, enabling the replenishment of the lysosomal population to sustain cellular metabolism and recycling functions, thereby ensuring cells can adapt to metabolic needs and stress (Yang and Tan 2023).

Transcription factor EB (TFEB) is a master regulator of lysosomal biogenesis and function, which orchestrates the expression of a wide array of genes involved in the generation of new lysosomes and the promotion of autophagy (Napolitano and Ballabio 2016).

The mechanistic target of rapamycin (mTOR) and calcineurin are central to the regulation of TFEB through their effects on TFEB's phosphorylation status (Napolitano and Ballabio 2016). When TFEB is phosphorylated, primarily at serine residues 211 and 142, it remains inactive in the cytosol. Conversely, dephosphorylation of TFEB triggers activation and translocation to the nucleus to promote the transcription of the Coordinated Lysosomal Expression and Regulation (CLEAR) gene network (Napolitano and Ballabio 2016). mTOR1, acting as a crucial nutrient sensor and cellular growth regulator, inhibits TFEB by phosphorylating it under nutrient‐rich conditions, preventing its nuclear translocation and activity. However, under conditions of starvation or lysosomal stress, mTOR1 activity is diminished, leading to reduced phosphorylation of TFEB.

In addition to amino acids and insulin, which are well‐established regulators of mTORC1 (Szwed et al. 2021), the pathway's activity is also modulated by cellular energy levels and stress signals. During oxidative stress, the regulation of mTORC1 is modulated by the cellular energy sensor AMP‐activated protein kinase (AMPK) and the AKT pathway, both of which respond to changes in cellular energy status and oxidative damage. In response to increased oxidative stress and decreased cellular energy load (as indicated by a high AMP:ATP ratio), AMPK is activated. Upon activation, AMPK suppresses mTORC1 activity both by activating the tuberous sclerosis proteins 1 and 2 (TSC1‐TSC2) complex and through the direct phosphorylation of Raptor (Inoki et al. 2003; Gwinn et al. 2008). The inactivation of Akt leads to reduced mTOR signaling primarily by removing its inhibitory action on the TSC1–TSC2 complex, thereby activating the TSC1–TSC2 complex (Menon et al. 2014).

The role of mTOR signaling in senescence is complex and somewhat paradoxical. On one hand, mTORC1 is known to be active during senescence, where it supports the translation of specific components of the SASP, such as IL‐6 (Carroll et al. 2017; Narita et al. 2011). On the other hand, the accumulation of dysfunctional cellular structures and the need to mitigate oxidative damage during senescence require enhanced lysosomal function and autophagy, processes typically associated with mTORC1 inactivation. This suggests that the cellular response leading to senescence involves a balance between mTORC1 inactivation to enhance autophagic and lysosomal degradation pathways via TFEB, which has been observed to be a protective factor during senescence (Curnock et al. 2023; Cui et al. 2024), and mTORC1 activation to promote the synthesis of SASP components.

In this study, we explored the dynamics of lysosomal quality control mechanisms during SIPS, with a particular focus on TFEB status. We aimed to delineate the differential signaling responses between the initial stress phase and the subsequent senescent state. We observed lysosomal damage during the stress phase, along with an increase in ROS levels during this phase as reported in our previous study (Wedel et al. 2020). This significant upregulation of ROS production could be associated with the activation of AMPK and inactivation of Akt observed during the stress phase. These alterations in AMPK and Akt activity played a crucial role in the suppression of mTOR signaling, a condition that enabled TFEB to translocate into the nucleus and initiate the transcription of genes involved in lysosomal biogenesis and the autophagic response. As a result, autophagy was activated and lysosomal damage repaired. During the senescent state, autophagy remained active and lysosomes showed no signs of damage. This was accompanied by the mitigation of ROS levels, as we have shown previously (Wedel et al. 2020), restoring the signaling balance involving AMPK and Akt, finally leading to the reactivation of mTOR while concurrently, TFEB activity was reduced. Our findings highlight the transient dependence on TFEB during the initial stress response to maintain cellular viability and facilitate the clearance of damaged organelles, thereby allowing cells to progress into senescence.

## 2 Results

### 2.1 Transient Lysosomal Dysfunction Is Observed During SIPS Induction

**FIGURE 1:**
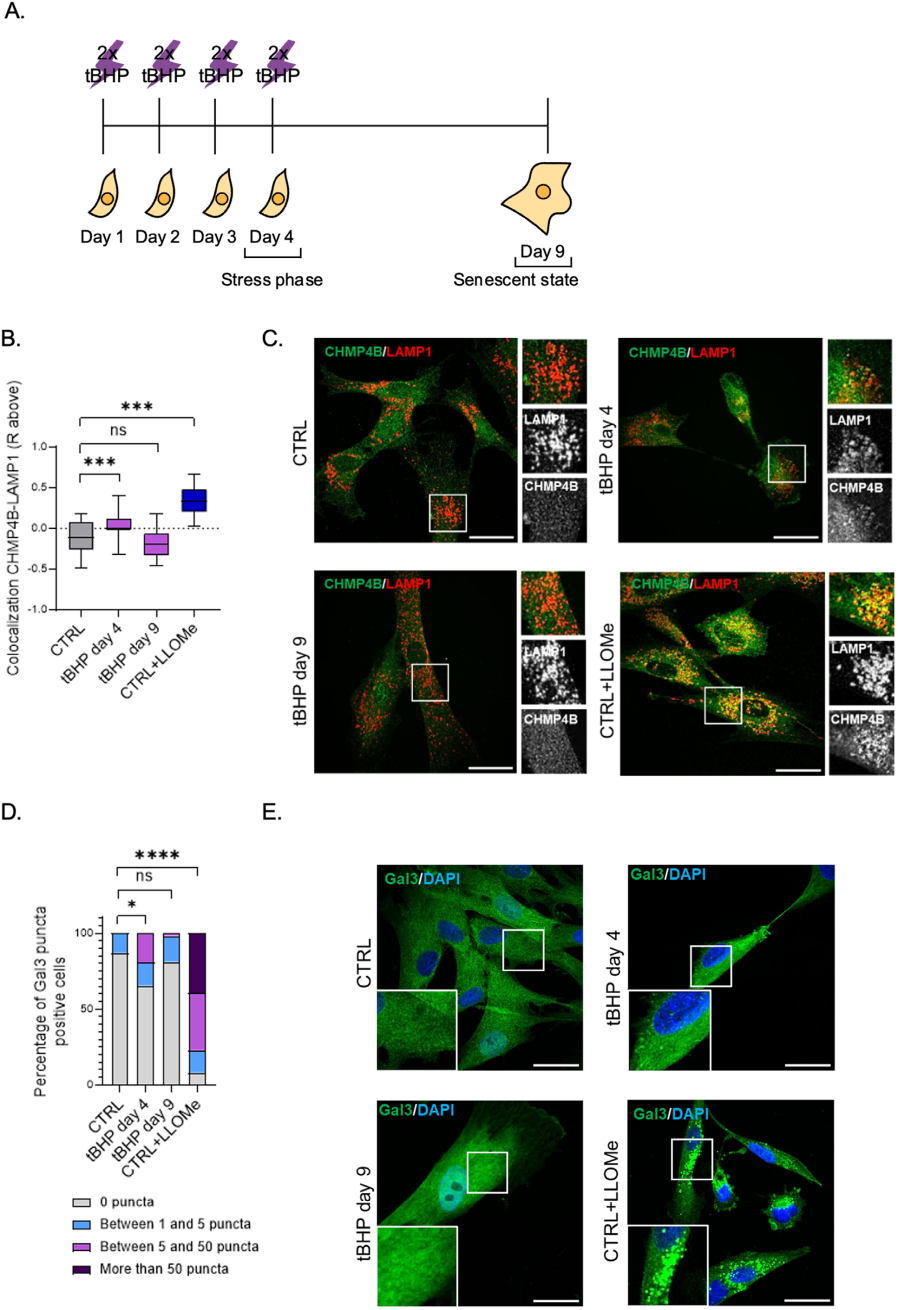
Lysosomal damage is increased by tBHP treatment. (A) Scheme showing tBHP SIPS induction on HDF. (B) Analysis of CHMP4B‐LAMP1 colocalization in tBHP‐stressed (tBHP day 4), tBHP‐senescent (tBHP day 9), and LLOMe treated HDFs. As positive control for lysosomal membrane damage, cells were treated with L‐Leucyl‐L‐leucine methyl ester (LLOMe), shown to induce lysosomal repair. (C) CHMP4B‐LAMP1 immunofluorescence representative pictures from control, tBHP day 4, tBHP day 9 and LLOMe treated HDFs. (D) Galectin3 puncta analysis from control, tBHP stress phase (day 4), tBHP senescent state (day 9) and LLOMe treated HDF. (E) Galectin3 immunofluorescence representative pictures from control, tBHP day 4, tBHP day 9 and LLOMe treated HDFs. Scale bars represent 30 μm. Data represents mean values ± SD, *N* = 3. In all graphics ns, Nonsignificant, *p < 0.05, **p < 0.01, ***p < 0.001, ****p < 0.0001.

We previously established a SIPS model of tert‐butyl hydroperoxide (tBHP)‐induced senescence in human dermal fibroblasts (HDF), in which we applied tBHP twice a day for four consecutive days (Wedel et al. 2020), under which conditions cells reach the senescent state on day 9 (Figure 1A). Given the critical role of lysosomes in cellular homeostasis, we aimed to further explore this model and study how lysosomal function and signaling are impacted during tBHP‐induced SIPS. To assess lysosomal dysfunction and quality control mechanisms during SIPS, cells were analyzed at day 4, representative for the stress phase, and day 9 where the senescent state was fully established (Wedel et al. 2020). Our initial analysis focused on evaluating the lysosomal repair mechanism via CHMP4B‐LAMP1 colocalization and by quantifying Galectin3 puncta. Our results indicated a transient enhancement in CHMP4B recruitment in stressed HDF but not in senescent HDF (Figure 1B,C). Furthermore, we observed a moderate and transient increase in Galectin3 puncta recruitment in stressed but not senescent HDF (Figure 1D,E).

We observed similar results in a previously established UVB SIPS model (Greussing et al. 2013) (Figure S1A). Our findings revealed a transient increase in CHMP4B recruitment in UVB‐stressed HDFs, but not in senescent HDFs (Figure S1B,C). Additionally, we observed a moderate and transient rise in Galectin3 puncta recruitment in UVB‐stressed HDFs, whereas this response was absent in senescent HDFs (Figure S1D,E).

### 2.2 Autophagic Activity is Increased During SIPS

**FIGURE 2:**
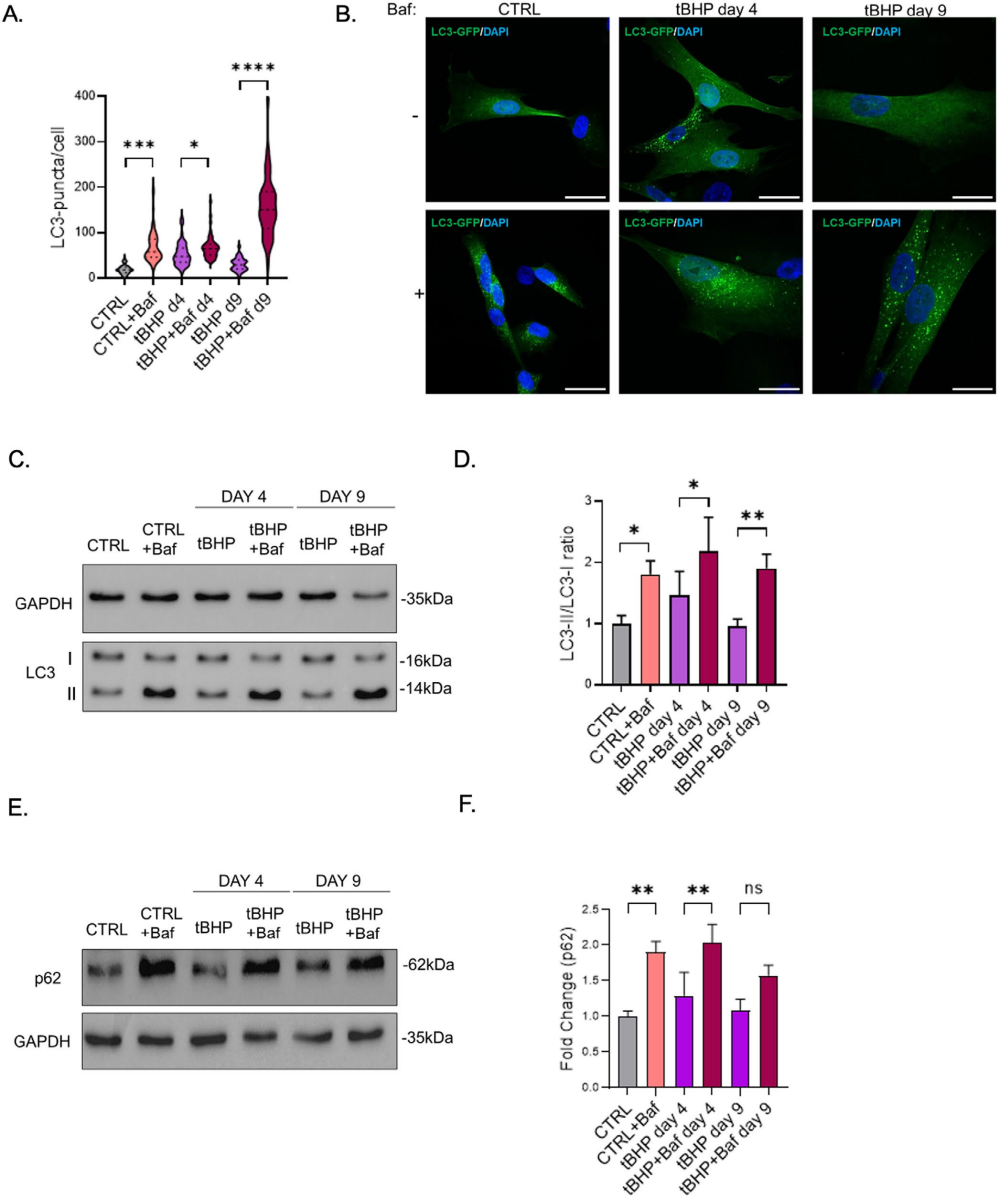
Autophagic flux increases in response to tBHP stress. (A) LC3‐puncta quantification from LC3‐GFP HDF in tBHP stress‐phase ± bafilomycin (day 4) and tBHP senescent state ± bafilomycin (day 9). (B) Representative pictures from LC3‐GFP tBHP stress‐phase ± bafilomycin (day 4) and tBHP senescent state (day 9). (C) LC3 Western blot image from tBHP stressed HDFs (day 4) and tBHP senescent HDFs (day 9) ±bafilomycin. (D) Densitometric quantification of LC3‐II/LC3‐I in tBHP stress‐phase (day 4) and tBHP senescent state (day 9) ± bafilomycin. (E) p62 Western blot image from tBHP stressed HDFs (day 4) and tBHP senescent HDFs (day 9) ± bafilomycin. (F) Densitometric quantification of p62 in tBHP stress‐phase (day 4) and tBHP senescent state (day 9) ± bafilomycin. Scale bars represent 30 μm. Data represents mean values ± SD, *N* = 3. In all graphics ns, Nonsignificant, *p < 0.05, **p < 0.01, ***p < 0.001, ****p < 0.0001.

To determine if lysosomal damage observed was affecting the capacity of the cells to degrade cellular components, we investigated whether autophagic flux was altered in response to tBHP treatment. During the tBHP stress phase, we observed an increase in the number of autophagosomes in comparison to control HDFs, but a partial impairment of the autophagic flux (Figure 2A,B), as reflected by a limited accumulation of autophagosomes following autophagy blocking by bafilomycin A1. These findings suggest that partial lysosomal dysfunction impedes the full functionality of the autophagic process. Autophagic flux was strongly increased in the senescent state, as evidenced by the increased number of LC3 puncta upon treatment with bafilomycin, supported by LC3 (Figure 2C,D) and p62 immunoblotting (Figure 2E,F). These findings reveal that autophagy is partially inhibited during the stress phase, likely as a result of tBHP‐induced damage, but fully recovered in the senescent state, suggesting that lysosomal activity was recovered when tBHP treatment was discontinued. Similarly, we previously demonstrated a strong enhancement of autophagic activity in our UVB‐induced senescence model, mostly in the stress phase (Cavinato et al. 2017). In this model, autophagic flux remained high until day 7 before returning to control levels by day 9.

### 2.3 TFEB Activity is Increased During the Stress Phase

**FIGURE 3:**
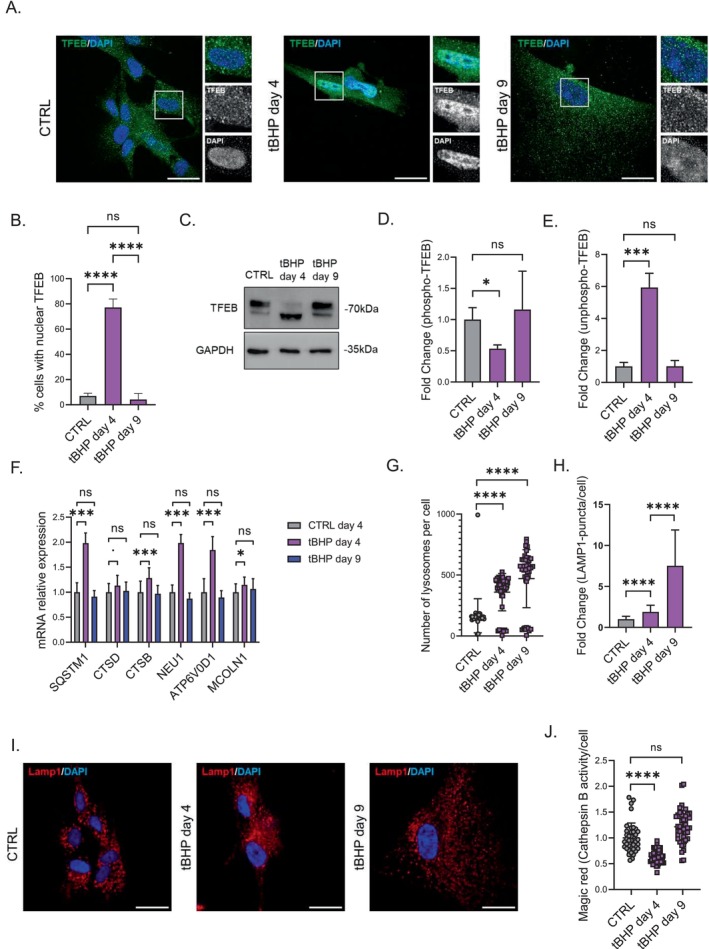
TFEB is active during tBHP stress‐phase but its activity diminishes during senescent state. (A) TFEB immunofluorescence representative pictures from control, tBHP‐stressed (day 4) and tBHP senescent (day 9) HDFs. (B) Quantification of nuclear TFEB percentages in control, tBHP stress phase (day 4) and tBHP senescent state (day 9). (C) TFEB western blot image in tBHP‐stressed (day 4) and tBHP‐senescent (day 9) HDFs. (D) Densitometric analysis of phosphorylated TFEB band during the stress phase (day 4) and senescent phase (day 9). (E) Densitometric analysis of unphosphorylated TFEB band during the stress phase (day 4) and senescent phase (day 9). (F) Relative mRNA expression of TFEB downstream effector genes *SQSTM1*, *CTSD*, *CTSD*, *NEU1*, *ATP6V0D1*, *MCOLN1*. (G) High content analysis of lysosomal numbers from control, tBHP day 4 and tBHP day 9 HDFs. (H) Comparative analysis of the fold change in LAMP1 puncta in LAMP1 immunofluorescence pictures among control, tBHP day 4 and tBHP day 9 HDFs. (I) LAMP1 immunofluorescence representative pictures from control, tBHP day 4 and tBHP day 9 HDFs. (J) Magic red fluorescence intensity as a measure of Cathepsin B activity on tBHP day 4 and tBHP day 9. Scale bars represent 30 μm. Data represents mean values ± SD, *N* = 3. In all graphics ns, Nonsignificant, *p < 0.05, **p < 0.01, ***p < 0.001, ****p < 0.0001.

Since TFEB is known to promote autophagy and lysosomal biogenesis, we explored TFEB activity in our models. During the tBHP‐induced stress phase, TFEB was translocated to the nucleus, indicating its activation. However, as cells transitioned to senescence, TFEB was observed mainly in the cytosol, indicating senescence‐associated TFEB inactivation (Figure 3A,B). Western blot analysis further revealed an increase in the unphosphorylated TFEB during the stress phase (Figure 3C–E), with phosphorylation levels of TFEB returning to baseline in tBHP‐induced senescent HDF (Figure 3C–E). Additionally, the transcriptional upregulation of several TFEB‐regulated genes during the stress phase but not during the senescent state was observed (Figure 3F). These findings demonstrate that TFEB was active during exposure to tBHP and inactivated as HDFs progressed to senescence.

In response to tBHP exposition, the number of lysosomes per cell was increased with a further increase of lysosomal numbers during the transition to senescence (Figure 3G–I). Lysosomal dysfunction was also evidenced by the reduced activity of cathepsin B, as observed in tBHP‐stressed HDF (Figure 3J). The activity of this enzyme was restored in tBHP‐induced senescent HDF, suggesting that the increase in lysosomal numbers may serve to counterbalance the compromised functionality of lysosomes during the stress phase.

To further understand TFEB dynamics, we also assessed TFEB activation in the UVB SIPS model. In UVB‐stressed HDFs, we observed a significant increase in nuclear TFEB localization on day 4, which returned to control levels by day 9 (Figure S2A,B). Western blot analysis confirmed an increase in both phosphorylated and unphosphorylated TFEB at day 4, while by day 9, these levels were indistinguishable from controls (Figure S2C–F). These data suggest that TFEB activation is a transient event during the UVB stress response, followed by its inactivation upon entry into senescence. Similarly, in the UVB model, lysosomal numbers were significantly increased on both day 4 and day 9 (Figure S2G,H).

### 2.4 mTOR is Inactive During the Stress Phase

Since TFEB activation was more pronounced in the tBHP model, we focused our study on TFEB dynamics in this model. Given mTOR's well established role as a regulator of TFEB activity (Napolitano and Ballabio 2016), we assessed the activation status of mTOR in the tBHP model, both during the stress phase and the senescent state.

**FIGURE 4:**
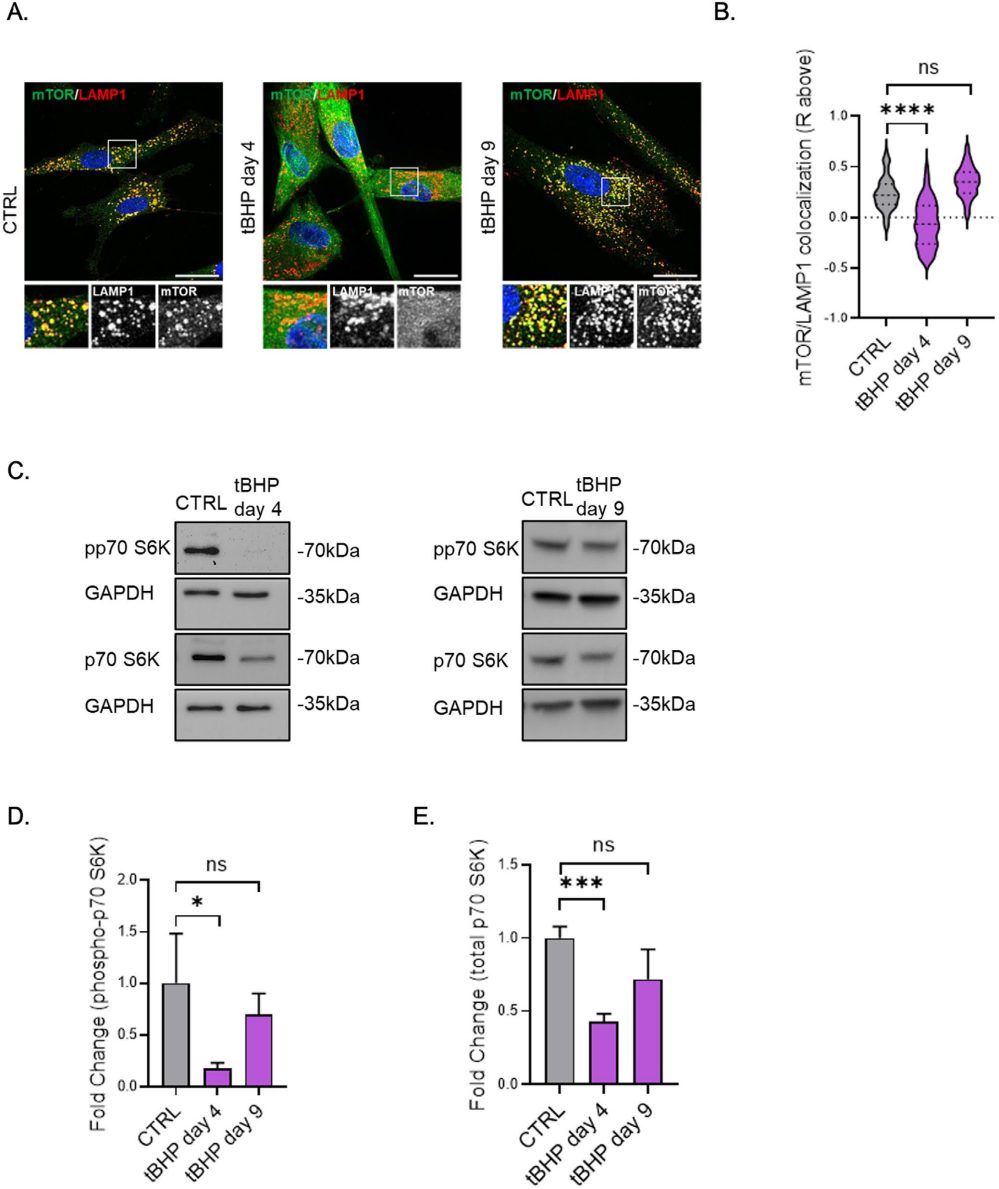
mTOR activity is diminished during tBHP stress phase but its activity is recovered during senescence. (A) mTOR‐LAMP1 immunofluorescence representative pictures from the stress phase (tBHP day 4) and senescent state (tBHP day 9). (B) Quantification of mTOR/LAMP1 colocalization during the stress phase (tBHP day 4) and senescence state (tBHP day 9). (C) phosphorylated‐p70S6K and total p70S6K western blot images from tBHP‐stressed (tBHP day 4) and tBHP senescent (day 9) HDFs. (D) Densitometric analysis of phosphorylated‐p70S6K from tBHP‐stressed (tBHP day 4) and tBHP senescent (day 9) HDFs. (E) Densitometric analysis from total‐p70S6K from tBHP‐stressed (tBHP day 4) and tBHP senescent (day 9) HDFs. Scale bars represent 30 μm. Data represents mean values ± SD, *N* = 3. In all graphics ns, Nonsignificant, *p < 0.05, **p < 0.01, ***p < 0.001, ****p < 0.0001.

At day 4, we observed a reduction in the colocalization of mTOR with lysosomes, whereas in the senescent state, mTOR colocalized with lysosomes (Figure 4A,B), suggesting that mTOR may be inactivated during the stress phase but is reactivated in senescent cells. To further validate this hypothesis, we examined the phosphorylation status of the canonical mTOR substrate, p70S6 kinase (p70‐S6K). Our observations revealed diminished phosphorylation during the stress phase (Figure 4C–E), with phosphorylation levels returning to normal in tBHP‐induced senescent HDFs (Figure 4C–E). Altogether, these results indicate a dynamic regulation of mTOR activity, which is inactive during the stress phase, aligning with the unphosphorylated TFEB status in tBHP‐stressed HDF. During the senescent state, mTOR appears to be active, correlating with the increased phosphorylation of TFEB.

### 2.5 Oxidative Stress Mediates mTOR Inactivation During SIPS Induction

To delve deeper into the reasons behind mTOR inactivity during the stress phase and considering that cells were cultured in full‐medium conditions and that tBHP is known to blunt the antioxidant response, we hypothesized that mTOR might be inactivated by oxidative stress under these conditions, a notion supported by existing literature (Heberle et al. 2014). Based on our observation that tBHP treatment leads to increased levels of both cytosolic and mitochondrial ROS (Wedel et al. 2020), we examined AMPK and Akt signaling pathways, both affected by ROS and recognized regulators of mTOR (Heberle et al. 2014).

**FIGURE 5:**
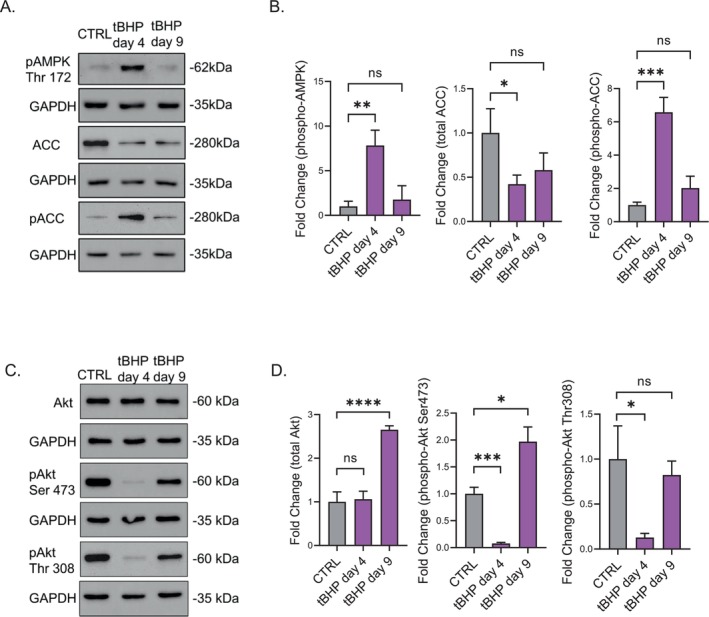
Activation of AMPK and inhibition of Akt pathways under tBHP‐induced stress. (A) phosphorylated AMPK (Threonine 172) (p‐AMPK), Acetyl Coa Carboxylase (ACC) and phosphorylated ACC (Serine 79) (p‐ACC) western blot images during the tBHP stress‐phase (tBHP day 4) and senescent state (tBHP day 9). (B) Densitometric analysis of p‐AMPK, total‐ACC, and p‐ACC in tBHP day 4 and tBHP day 9. (C) total Akt, phosphorylated‐Akt (Serine 473) (p‐Akt Ser 473), and phosphorylated‐Akt (Threonine 308) (p‐Akt Thr 308) western blot image from tBHP day 4 and tBHP day 9. (D) Densitometric analysis from total‐Akt, p‐Akt Ser 473 and p‐Akt Thr 308 in tBHP day 4 and tBHP day 9. Data represents mean values ± SD, *N* = 3. In all graphics ns, Nonsignificant, *p < 0.05, **p < 0.01, ***p < 0.001, ****p < 0.0001.

AMPK activity was elevated during the stress phase in tBHP‐induced SIPS, as indicated by enhanced phosphorylation of AMPK at Threonine 172 (Figure 5A,B) and increased phosphorylation of its substrate, Acetyl‐CoA Carboxylase (ACC), at Serine 79 (Figure 5A,B). These phosphorylation events diminished in tBHP‐induced senescent HDFs (Figure 5A,B), showing that AMPK activation is no longer present once the cells achieve the senescent state. These findings suggest that AMPK activation plays a pivotal role in cellular adaptation to oxidative stress by tBHP exposition.

Akt activity was diminished during the stress phase, as demonstrated by decreased phosphorylation at Serine 473 and Threonine 308 (Figure 5C,D). By day 9, there was a recovery in Akt signaling, with senescent cells exhibiting elevated levels of Akt phosphorylation and overall Akt protein levels (Figure 5C,D), indicating a dynamic modulation of Akt signaling in response to cellular stress and senescence. These observations suggest a regulatory interplay between AMPK and Akt pathways, where both pathways may autoregulate and crosstalk with each other (Zhao et al. 2017), contributing to the intricate balance between cellular stress responses and the senescence program.

### 2.6 TFEB is Essential for Senescence Induction but Not for Its Maintenance

**FIGURE 6:**
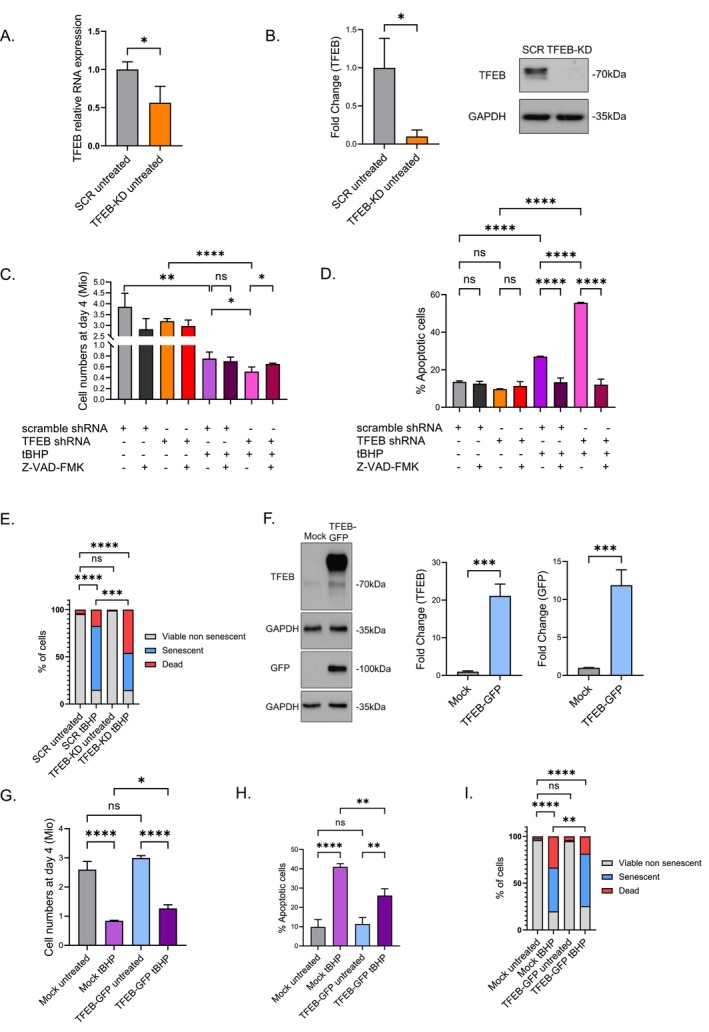
Apoptotic increase in TFEB‐KD HDFs subjected to tBHP treatment. (A) TFEB relative mRNA expression in TFEB‐KD HDFs. (B) TFEB western blot images from TFEB‐KD HDFs. Densitometric analysis of TFEB from TFEB‐KD HDFs. (C) Cell numbers (in millions) of SCR and TFEB‐KD HDFs treated with tBHP, with and without the caspase inhibitor Z‐VAD‐FMK. (D) Quantification of apoptotic cells by AnnexinV‐PI in SCR and TFEB‐KD HDFs subjected to tBHP with and without Z‐VAD‐FMK. (E) Percentage of dead HDFs, viable nonsenescent HDFs and senescent HDFs as a measure of SA‐β‐gal positive cells at day 9. (F) TFEB and GFP western blot images from TFEB‐GFP HDFs. Densitometric analysis of TFEB and GFP from TFEB‐GFP HDFs. (G) Cell numbers (in millions) of Mock and TFEB‐GFP HDFs treated with tBHP. (H) Quantification of apoptotic cells by AnnexinV‐PI in Mock and TFEB‐GFP HDFs subjected to tBHP. (I) Percentage of dead HDFs, viable nonsenescent HDFs and senescent HDFs as a measure of SA‐β‐gal positive cells at day 9. Data represents mean values ± SD, *N* = 3. In all graphics ns: Nonsignificant, *p < 0.05, **p < 0.01, ***p < 0.001, ****p < 0.0001.

To assess a potential role of TFEB in supporting cell viability during the stress phase associated with the induction of SIPS, TFEB expression was impaired by shRNA‐mediated knockdown (TFEB‐KD) (Figure 6A,B). Following exposure to tBHP treatment, TFEB‐KD HDFs exhibited decreased cell numbers in comparison to SCR HDFs (Figure 6C), supporting an essential role of TFEB for HDF survival under stress conditions. The percentage of apoptotic cells, revealed by Annexin V/PI staining, was increased in tBHP‐treated TFEB‐KD HDFs (Figure 6D). The induction of cell death was reversed with the use of a pan‐caspase inhibitor (Figure 6C,D). This evidence underscores the critical importance of TFEB in maintaining cell viability under challenges induced by oxidative stress.

Subsequently, we examined the development of the senescent phenotype in TFEB‐KD cells. As a result of the greater percentage of TFEB‐KD HDFs that died upon tBHP treatment, the number of cells undergoing senescence was diminished (Figure 6E).

To further investigate the role of TFEB in cell survival under oxidative stress, we overexpressed TFEB (TFEB‐GFP) in HDFs (Figure 6F). Upon tBHP treatment, TFEB‐GFP HDFs exhibited a significant increase in cell numbers at day 4 in comparison to mock‐transfected cells (Figure 6G), indicating that TFEB overexpression enhances cell survival under stress conditions. Consistently, Annexin V/PI staining revealed a reduction in apoptosis in TFEB‐GFP HDFs following tBHP exposure (Figure 6H). Despite this increased resistance to cell death, TFEB‐GFP‐expressing cells were able to progress into senescence, as indicated by an elevated proportion of senescent cells at later time points (Figure 6I).

These findings suggest that TFEB is crucial for supporting cell survival under adverse stimuli, allowing the cells to transition into the senescent state, suggesting that TFEB plays a major role in the initial stress response rather than in the maintenance of the senescent state.

### 2.7 TFEB Modulates Cellular Response to tBHP Stress

**FIGURE 7:**
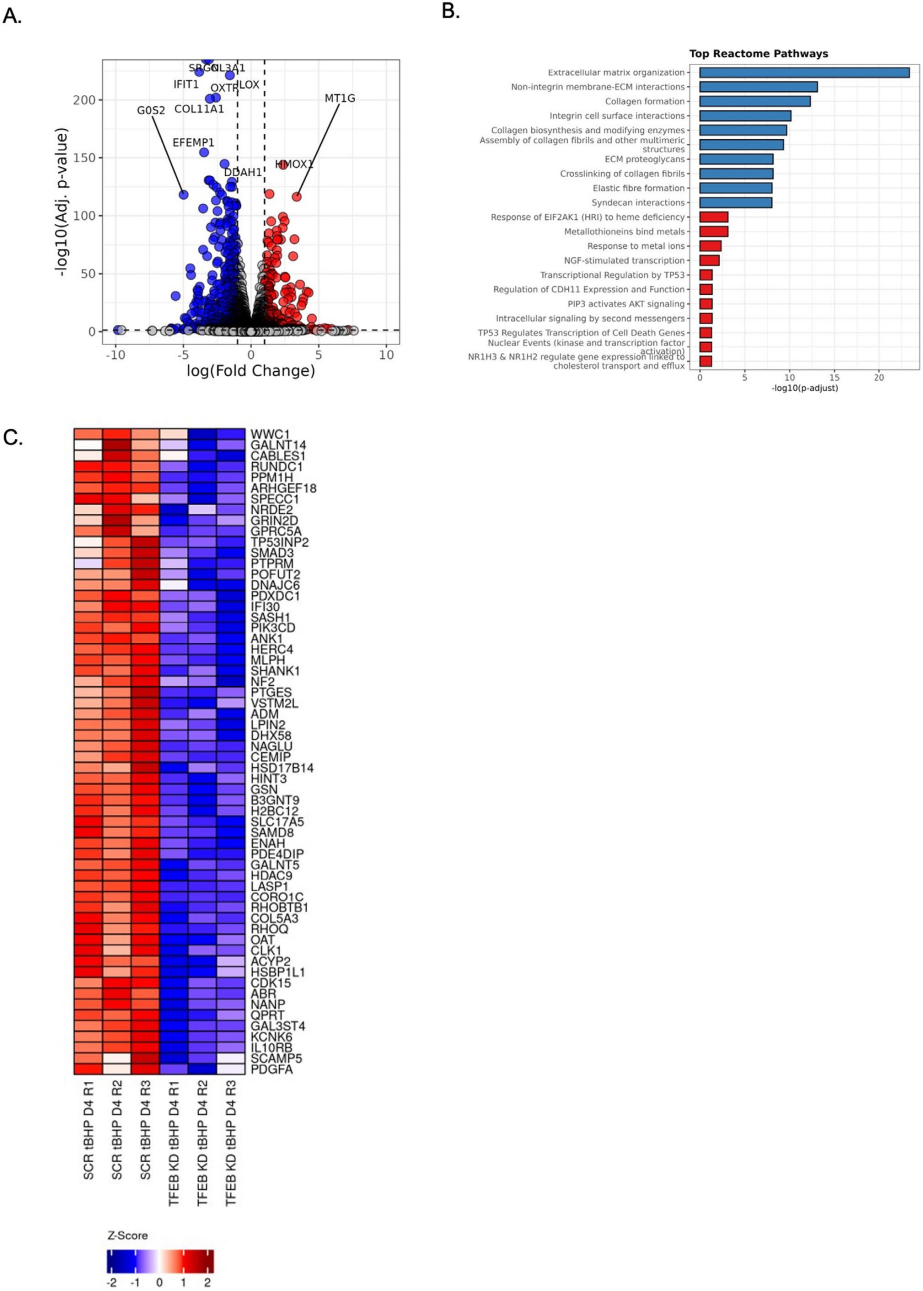
Transcriptomic Analysis of TFEB‐KD vs SCR HDFs. (A) Volcano plot of DEGs in TFEBKD vs. SCR. Each dot represents a gene; upregulated (logFC > 1, *p*adj < 0.05) in red, downregulated (logFC < −1, *p*adj < 0.05) in blue, and non‐significant in black/gray. Dashed lines mark significance thresholds. Labeled genes are highly differentially expressed. (B) Over‐representation analysis (ORA) of Reactome pathways using differentially expressed genes (DEGs) (*padj < 0.05, |log_2_FC| > 0.36). ORA was performed separately for upregulated (red) and downregulated (blue) genes. The bar plot shows the top significant pathways (*p*adj < 0.05) ranked by –log_10_(adjusted *p*‐value). (C) Heatmap of common downregulated genes that are also TFEB targets. Expression values are shown as *Z*‐scores from normalized count data. Rows represent genes, and columns represent samples. Hierarchical clustering is applied to genes.

To further explore the role of TFEB during the stress conditions, we performed an RNA‐seq comparing SCR and TFEB‐KD HDFs at tBHP day 4. Differential expression analysis revealed significant transcriptional changes upon TFEB knockdown, with a large subset of genes upregulated or downregulated in TFEB‐KD cells compared to SCR controls (Figure 7A). To evaluate pathways regulated in response to depletion of TFEB, we performed an Over‐Representation Analysis (ORA) of Reactome pathways using differentially expressed genes (DEGs) (Figure 7B). ORA revealed that pathways related to extracellular matrix organization, integrin interactions, and collagen biosynthesis were significantly downregulated in TFEB‐KD cells (blue), while pathways associated with heme deficiency, metal ion responses, and p53‐regulated cell death genes were enriched in the upregulated gene set (red). This suggests that TFEB depletion disrupts extracellular remodeling and stress adaptation pathways, potentially exacerbating cellular damage.

Additionally, a subset of common downregulated genes in TFEB‐KD cells was identified as direct TFEB targets (Figure 7C). The heatmap illustrates a consistent reduction in the expression of these genes in TFEB‐KD HDFs, reinforcing the role of TFEB as a transcriptional regulator of stress‐responsive pathways. Notably, we did not identify any pathways specifically associated with senescence (data not shown), suggesting that TFEB primarily regulates the acute stress response rather than directly influencing the senescence program.

### 2.8 TFEB Activation Promotes Cell Survival During SIPS Induction Without Affecting Senescence Progression

**FIGURE 8:**
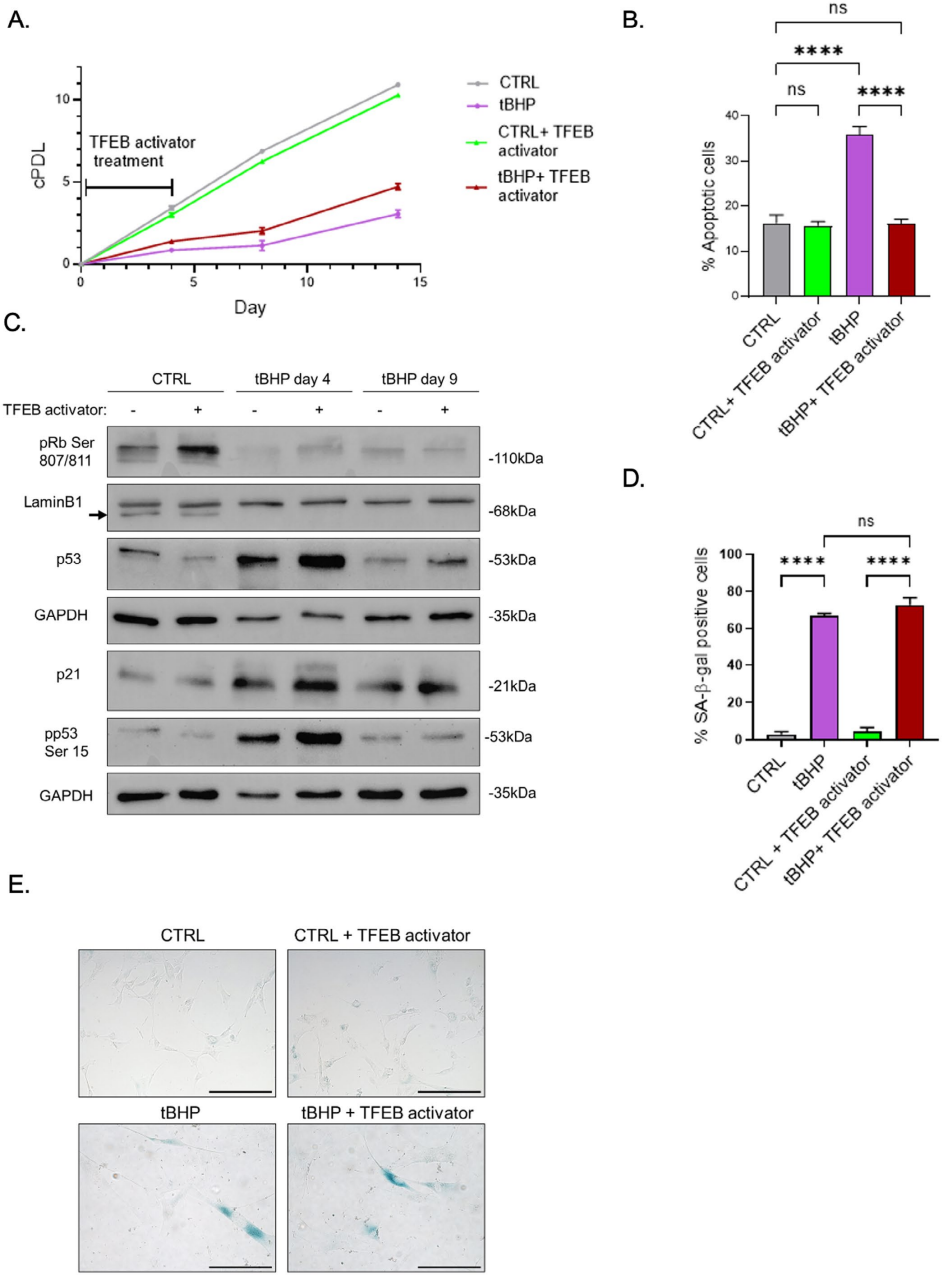
TFEB modulates cellular response to oxidative stress. (A) Growth curve showing cumulative population doublings (cPDL) in control and tBHP treated HDFs ± TFEB activator. (B) Quantification of apoptotic cells by AnnexinV‐PI in control and tBHP treated HDFs ± TFEB activator. (C) Western blot images of senescence markers in control and tBHP HDFs ± TFEB activator. (D) Quantification of SA‐β‐galactosidase‐positive cells in control and tBHP HDFs ± TFEB activator. (E) SA‐β‐gal staining pictures from control and tBHP treated HDFs (day 9) ± TFEB activator. Scale bars represent 20 μm. Data represents mean values ± SD, *N* = 3. In all graphics ns, Nonsignificant, *p < 0.05, **p < 0.01, ***p < 0.001, ****p < 0.0001.

To explore the role of TFEB in senescence induction, we tested whether its activation by a small molecule affects cellular outcomes under tBHP exposure. TFEB activation increased cumulative population doublings (Figure 8A) and reduced the number of apoptotic cells (Figure 8B). It also influenced p53 Ser 15 phosphorylation on day 4 (Figure 8C), suggesting a potential role in enhancing cellular repair under stress conditions. However, no significant changes were observed in senescence markers on day 9 (Figure 8C, Figure S3A–E) or in SA‐β‐gal staining (Figure 8D,E), confirming that TFEB activation primarily enhances cellular survival without affecting senescence.

## 3 Discussion

In this study, we explored mechanisms of lysosomal quality control during cellular senescence induced by chronic oxidative stress. Our findings suggest that lysosomal activity plays a crucial role in promoting cell survival under stress conditions, enabling surviving cells to develop the senescence phenotype, in agreement with our previous finding that blocking autophagy in UV‐irradiated HDF changed cell fate from senescence to apoptotic cell death (Cavinato et al. 2017). Upon tBHP treatment, we observed the emergence of dysfunctional lysosomes, characterized by the appearance of CHMP4B punctae and Galectin3 punctae, indicators for the transient activation of lysosome repair pathways. We also observed an increase in lysosomal numbers, further increasing the autophagic capacity of the cells. A compensatory increase in lysosomal biogenesis has been previously documented in the context of OIS and other forms of senescence (Curnock et al. 2023).

In tBHP‐treated cells, we observed inactivation of Akt and the concurrent activation of AMPK, leading to mTOR inactivation (Heberle et al. 2014). In turn, mTOR inactivation triggered dephosphorylation, nuclear localization, and activation of the transcription factor TFEB, which played a crucial role in promoting cellular survival under stress conditions. In this scenario, TFEB activation was a consequence of lysosomal damage, explaining why at day 4, partially impaired autophagy coexisted with activated TFEB. TFEB activation contributed to the adaptation of cells to stress, allowing them to maintain homeostasis and transit into a stable senescent state. Discontinuing tBHP treatment brought AMPK and Akt activity back to baseline, leading to the reactivation of mTOR, followed by the phosphorylation and subsequent inactivation of TFEB. At the same time, lysosomal damage ceased, and activated TFEB restored the lysosomal network, assisted by lysosomal repair mechanisms (Galectin and CHMP4B). Therefore, on day 9, we observed increased autophagy coexisting with inactive TFEB. Taken together, our findings suggest that precisely timed activation and subsequent inactivation of TFEB pave the way to cellular senescence in tBHP‐treated cells.

Previous research has highlighted the importance of lysosomal repair in maintaining the integrity of lysosomes, which is crucial for their proper function (Yang and Tan 2023). Additionally, lysosomal damage leads to an increase in lysosomal biogenesis, which, through TFEB, the master regulator of lysosomal biogenesis, supports autophagy (Saftig and Puertollano 2021). We propose a working hypothesis where oxidative stress induces lysosomal damage and modulates AMPK and Akt signaling, leading to mTOR suppression (Heberle et al. 2014)—a key step for TFEB activation (Napolitano and Ballabio 2016). TFEB activation can be driven by lysosomal damage (Nakamura et al. 2020), by calcineurin‐mediated dephosphorylation under stress conditions (Medina et al. 2015), and also by accumulation of ROS (Martina et al. 2021). To delineate the precise consequences of these events, future studies should employ systematic, combinatorial approaches that selectively inhibit or activate these key regulatory pathways—including AMPK, Akt, mTOR, and calcineurin—thus elucidating the distinct contributions and interplay of each in TFEB regulation.

Upon cessation of tBHP treatment, HDF transitioned into the senescent state, and TFEB was inactivated, probably reflecting a decreased demand for damage repair. It is possible that the generation of free amino acids (Narita et al. 2011) by enhanced autophagy contributes to the reactivation of mTOR and subsequent TFEB inactivation. Another hypothesis suggests that successful autophagy of damaged organelles could reduce ROS production, thereby facilitating the restoration of Akt signaling and diminishing AMPK activity (Heberle et al. 2014). These changes would permit the reactivation of mTOR. In fact, mTOR is known to be active during senescence (Laberge et al. 2015; Herranz et al. 2015), where it promotes the synthesis of SASP factors. More work will be required to delineate the complex interplay of these factors to shape the senescence phenotype in tBHP treated cells.

TFEB‐depleted cells exhibited a markedly diminished capacity to manage tBHP‐induced stress, leading to apoptotic cell death. This phenomenon underscores the critical need for stressed cells to augment lysosomal biogenesis and enhance autophagy as essential responses to induce a senescence phenotype. Additionally, it is plausible that TFEB may promote the expression of antioxidant genes, offering another layer of protection (Park et al. 2019; Jeong et al. 2022). TFEB serves as a protective mechanism, safeguarding cellular survival under adverse conditions by activating its transcriptional program, which includes the CLEAR network, thereby enhancing autophagic processes (Napolitano and Ballabio 2016). The role of TFEB in promoting lysosomal biogenesis and autophagy elucidates its pivotal function in cellular defense mechanisms, highlighting the necessity of these processes for maintaining cellular integrity and function during stress. Transcriptomic analysis in dermal fibroblasts reported here (Figure 7B) revealed a decrease in collagen‐related gene expression, collagen matrix organization, and elastic fiber formation in TFEB‐KD fibroblasts, suggesting that TFEB is essential for preserving fibroblast function, potentially linking its activity to skin aging.

Our findings suggest that TFEB activation is important to ensure cellular survival during SIPS induction. Following the establishment of senescence, however, TFEB appears to be dispensable, aligning with our observations that its activation does not affect the ultimate progression toward a senescent phenotype. TFEB has been reported to be active during SIPS and OIS (Curnock et al. 2023; Urbanelli et al. 2014), while other authors only found TFEB active during the early phases of senescence progression (Cho and Hwang 2012) or observed reduced TFEB activity during senescence (Gianfranceschi et al. 2016). A broader investigation is crucial for a comprehensive understanding of the role of TFEB across different stress contexts and to elucidate if TFEB activity differs between the stress phase and the senescence state in other models.

Moreover, considering the role of TFEB in promoting cellular survival during senescence induction, pharmacological inhibition of TFEB may enhance the elimination of cells undergoing SIPS during the stress phase. However, no widely accepted TFEB inhibitors are currently available. Identifying or developing such inhibitors could provide a novel strategy for selectively targeting stressed cells before they enter a senescent state, potentially mitigating their long‐term impact on tissue function.

## 4 Materials and Methods

### 4.1 Cell Culture

HFF‐2 HDF were acquired from ATCC (Manassas, VA, SCRC‐1042). Cells were cultured in DMEM low glucose media (Sigma, D5921), supplemented with 10% heat inactivated fetal bovine serum (FBS) (Gibco, 10270‐106), glutamine 4 mM (Sigma, G7513) and penicillin/streptomycin 1% (Sigma, P4333) in a humid incubator at 37°C and 5% CO_2_. Cell numbers were quantified utilizing a CASY counter (OMNI Life Science, Bremen, Germany). The cPDLs were determined using the equation PDL = (ln*F* − ln*I*)/ln2, where *F* represents the number of cells at the end of a passage, and *I* denotes the initial number of seeded cells at the beginning of the passage.

For SIPS models (tBHP and UVB), HDFs were used at passages 9–11.

HDF treated with tBHP were plated at a density of 6 × 10^5^ cells in 10 cm dishes, while control fibroblasts were seeded at a density of 3 × 10^5^ cells. These cells underwent incubation in DMEM media supplemented with 40 μM tBHP for 1 h, twice daily, over a span of four consecutive days, as outlined in reference (Wedel et al. 2020).

For UVB treatment (Greussing et al. 2013), irradiated fibroblasts were seeded in 10 cm plates at a density of 6 × 10^5^ cells, while control fibroblasts were seeded at 3 × 10^5^ cells. The cells were washed twice with Hank's balanced salt solution (HBSS, Sigma, Darmstadt, Germany, H8264) and covered with 2.5 mL of HBSS. They were then exposed to UVB irradiation at 0.05 J/cm^2^ using a Bio‐Sun System (Vilver Lourmat) twice daily for four consecutive days.

TFEB activator 1 (MedChemExpress, HY‐135825) was administered at a concentration of 1 μM throughout the 4‐day duration of the tBHP treatment.

### 4.2 Immunoblotting

Following two washes in cold PBS (Pan‐Biotech, P04‐36500), cells were collected by scraping, and protein extraction was performed using a radioimmunoprecipitation assay (RIPA) buffer enriched with protease inhibitors, including 50 mM NaF, 2 μg/mL Aprotinin, 1 mM PMSF, and 1 mM activated Sodium Orthovanadate.

The BCA protein assay kit (Pierce BCA Protein Assay Kit, Thermo, 23225) was used to determine the concentrations of protein lysates, with 20 μg of protein subsequently subjected to separation via SDS‐PAGE electrophoresis. Following separation, proteins were transferred onto a PVDF membrane (Immun‐Blot, Bio‐Rad, 1620177) using a 1X transfer buffer that included 10% methanol. This process was carried out in a wet chamber maintained at 4°C, with the voltage fixed at 100 V for 1 h and 10 min. To block the membranes, a 5% nonfat milk solution (Sigma, 70166) in TBS‐T (with 0.01% Tween 20) was employed for 1 h at room temperature, followed by an overnight incubation with the designated primary antibodies: LC3 A/B (#4108S, Cell Signaling), p62 (#ab155686, Abcam), TFEB (#4240, Cell Signaling), Phospho‐p70 S6 Kinase (Thr389) (#9205, Cell Signaling), p70 S6 Kinase (#9202, Cell Signaling), Phospho‐AMPKα (Thr172) (#2535, Cell Signaling), Phospho‐Acetyl‐CoA Carboxylase (Ser79) (#3661, Cell Signaling), Acetyl‐CoA Carboxylase (#3662, Cell Signaling), Akt (#9272, Cell Signaling), Phospho‐Akt (Ser473) (#9271, Cell Signaling), Phospho‐Akt (Thr308) (#9275, Cell Signaling), Phospho‐Rb Ser807/811 (#9308, Cell Signaling), Lamin B1 (#ab16048, Abcam), p53 (DO‐1) (sc‐126, Santa Cruz), Phospho‐p53 Ser15 (#9284, Cell Signaling), p21 Waf1/Cip1 (12D1) (#2947, Cell Signaling), GFP (D5.1) (#2956, Cell Signaling), GAPDH (0411, sc‐47,724, Santa Cruz).

The membranes were incubated for 1 h at room temperature with HRP‐conjugated secondary antibodies. For visualization, the HRP substrate (Immobilion Western, Millipore, WBKLS0500) was applied, and images were captured using the ChemiDoc Imaging System (Bio‐Rad). Densitometry analysis was performed with ImageJ software, employing GAPDH as the normalization reference.

### 4.3 Quantitative Real‐Time PCR

RNA was isolated using the RNeasy Mini kit (Qiagen, 74,004), and cDNA was synthesized using the High‐Capacity cDNA Reverse Transcription Kit (Thermo, 4368814) adhering to the manufacturer's guidelines. The AceQ Universal SYBR Green qPCR Master Mix (Vazyme, Q511‐02) was employed to conduct real‐time PCR in triplicate for each sample, using the QuantStudio 7 Flex Real‐Time PCR System (Thermo, 4485701). Primers were acquired from Eurofins Genomics, being the sequences from 5′ to 3′: SQSTM1 FW primer (GCA CCC CAA TGT GAT CTG C), SQSTM1 RE primer (CGC TAC ACA AGT CGT AGT CTG G), CTSD FW primer (TGC TCA AGA ACT ACA TGG ACG C), CTSD RE primer (CGA AGA CGA CTG TGA AGC ACT), CTSB FW primer (GAG CTG GTC AAC TAT GTC AAC A), CTSB RE primer (GCT CAT GTC CAC GTT GTA GAA GT), NEU1 FW primer (GGA GGC TGT AGG GTT TGG G), NEU1 RE primer (CAC CAG ACC GAA GTC GTT CT), ATP6V0D1 FW primer (TTC CCG GAG CTT TAC TTT AAC G), ATP6V0D1 RE primer (CAA GTC CTC TAG CGT CTC GC), MCOLN1 FW primer (TTC GCC GTC GTC TCA AAT ACT), MCOLN1 RE primer (CTC TTC CCG GAA TGT CAC AGC), B2M FW primer (GAA TTC ACC CCC ACT GAA AA), B2M RE primer (CTC CAT GAT GCT GCT TAC A). Normalization was done using the B2M housekeeping gene.

### 4.4 Stable Knockdown of TFEB

Lentiviral particles were produced by transfection of HEK293T cells with PLKO shTFEB (NM_007162.1‐1037s1c1) from Sigma in combination with the psPAX2 (Addgene, #12260), pMD2G/VSVG (Addgene, #12259) packaging plasmids using Lipofectamine LTX transfection reagent (Invitrogen). HDFs were infected with the lentiviral vector carrying scramble shRNA or TFEB shRNA, and beginning 2 days postinfection, cells were selected with puromycin.

### 4.5 Stable TFEB‐GFP Expression

Lentiviral particles were produced by transfection of HEK293T cells with the inducible overexpression plasmid for TFEB‐GFP (Settembre et al. 2011) (doxycycline‐inducible) in combination with the psPAX2 (Addgene, #12260) and pMD2.G/VSVG (Addgene, #12259) packaging plasmids using Lipofectamine LTX transfection reagent (Invitrogen). HDF were infected with the lentiviral vector carrying TFEB‐GFP or the mock control plasmid (Unterluggauer et al. 2008). Two days postinfection, cells expressing TFEB‐GFP were selected with puromycin, while mock‐infected cells were selected with blasticidin.

### 4.6 Stable LC3‐GFP Expression

Expression vectors containing the human LC3B gene fused to the 5' end of the GFP gene, along with lentiviral particles incorporating these vectors, were produced according to the method outlined by (Cavinato et al. 2017). HDFs expressing LC3‐GFP were subsequently created through lentiviral transduction. Images were taken with the Cell Voyager CV1000 Yokogawa confocal microscope (Visitron Systems) and LC3‐puncta quantification was performed using ImageJ software, analyzing at least 50 cells per group.

### 4.7 SA‐β‐Gal Cytochemistry Assay

After being cultivated on six‐well plates, cells were washed three times with PBS and underwent fixation with a solution of 2% formaldehyde and 0.4% glutaraldehyde in PBS. Subsequent to another three PBS washes, the cells were covered with a staining solution at pH 6.0, consisting of 150 mM NaCl, 2 mM MgCl2, 5 mM potassium ferricyanide, 5 mM potassium ferrocyanide, 40 mM citric acid, 12 mM sodium phosphate, and 1 mg/mL 5‐bromo‐4‐chloro‐3‐indolyl‐β‐D‐galactoside (X‐gal), and then incubated for 24 h at 37°C in a CO_2_‐free environment. To stop the reaction, the cells were washed three times with PBS. SA‐β‐gal positive cells were identified using a Nikon eclipse TE300 light microscope.

### 4.8 Immunofluorescence

For TFEB immunostaining, HSF were cultured on glass coverslips and fixed with 4% paraformaldehyde (PFA) at room temperature for 20 min, followed by three PBS washes. Permeabilization of these cells was achieved with a PBS solution containing 0.1% sodium citrate and 0.3% Triton X‐100. Blocking was conducted using 1% bovine serum albumin (BSA) in PBS. The cells were then incubated overnight at 4°C with the primary antibody against TFEB (#4240, Cell Signaling). Following multiple PBS washes, incubation with the secondary antibody (Alexa Fluor 488‐conjugated anti‐rabbit antibody, #A27034, ThermoFisher) was performed.

For the immunostaining of CHMP4B/LAMP1, GAL3, and mTOR/LAMP1, cells were cultured on glass coverslips and fixed using the previously described method. These cells were subjected to a gentle permeabilization and blocking with a saponin based buffer to preserve the integrity of the lysosomal membrane. The coverslips were then incubated with the appropriate primary antibody for 1 h at room temperature (CHMP4B, #42466, Cell Signaling; LAMP1, #14‐1079‐80, ThermoFisher; Galectin3, #14979‐1‐AP, Proteintech; mTOR, #2983, Cell Signaling). Following several washes, the cells were incubated with the respective secondary antibody (Alexa Fluor 488‐conjugated anti‐rabbit, #A27034, ThermoFisher; Alexa Fluor 546‐conjugated anti‐mouse, #A‐11003, ThermoFisher).

Cells were observed in a Cell Voyager CV1000 Yokogawa confocal microscope (Visitron Systems, Germany) and image analysis was done using ImageJ software, analyzing at least 50 cells per group.

### 4.9 High Content Image Analysis

The day before the experiment, cells were plated in 96‐well plates. To evaluate Cathepsin B activity, cells underwent staining with Magic Red (MR‐CtsB; 938, Immunochemistry Technologies) following the manufacturer's instructions. Images were captured using a confocal automated microscope (Opera High Content System; Perkin‐Elmer), with a minimum of 10 fields obtained for each well. Subsequently, cells were fixed and incubated overnight at 4°C with a primary antibody for LAMP1 (#14‐1079‐80, ThermoFisher), then washed with PBS and incubated with an appropriate secondary antibody. Images were again taken using the confocal automated microscope. For the analysis of Cathepsin B activity and the evaluation of lysosomal numbers and positioning, ad hoc‐designed scripts to calculate the red fluorescence intensity normalized to the total number of LAMP1 spots were employed.

### 4.10 Determination of Cell Death With AnnexinV/PI

Apoptotic and necrotic cells were identified using Annexin V/PI staining, adhering to the guidelines provided by the manufacturer (FITC Annexin V Apoptosis Detection Kit I, BD Pharmingen, Vienna, Austria). The detection of fluorescence signals was conducted with a BD FACS Canto II flow cytometer. The proportion of apoptotic cells was determined by adding together the percentages of necrotic (Annexin V‐negative/PI‐positive), early apoptotic (Annexin V‐positive/PI‐negative), and late apoptotic (Annexin V‐positive/PI‐positive) cells.

Starting from day 1 of tBHP treatment, cells were treated with a 20 μM concentration of the Pan‐caspase inhibitor Z‐VAD‐FMK (Promega, G7231), continuing until day 4, when the assessment of cell death rates was conducted.

### 4.11 RNA‐Seq Analysis

RNA samples were prepared using the Monarch Total RNA Miniprep kit (#T2010S, New England BioLabs), and sequencing was done by Azenta. RNA‐seq analysis was performed using the nf‐core RNA‐seq pipeline (available at DOI: 10.5281/zenodo.1400710). Reads were trimmed and aligned to the hg38 GENCODE reference using STAR (Dobin et al. 2013) and Salmon (Patro et al. 2017). Differential expression analysis was conducted with DESeq2 (Love et al. 2014), selecting significant DEGs based on *p*adj lower than 0.05 and absolute log_2_(FC) higher than 0.36. ORA and enrichment visualization were performed using Reactome pathways via the ReactomePA package (Yu and He 2016). TFEB target genes were obtained from TFEBexplorer (De Cegli et al. 2022). All analyses and visualizations were performed in R (R version 4.4.1).

### 4.12 Statistics

Each experiment was conducted with at least three independent biological replicates. Data are expressed as the mean ± standard deviation. Statistical significance between groups was assessed using a *t*‐test for comparisons between two conditions or a one‐way ANOVA for experiments involving more than two conditions. The significance levels are represented in the figures as follows: ns (not significant), *p < 0.05, **p < 0.01, ***p < 0.001, and ****p < 0.0001.

## Author Contributions

L.G.‐N., P.J.‐D., and M.C. designed the study and the experiments to be performed with input from M.E.G.D.A., J.M., L.A.H., A.B.L.G.‐N. performed the experiments and analyzed the data, with V.K. assisting in some experiments. P.M.‐L. conducted the bioinformatic analysis. M.C. supervised the work. L.G.‐N., P.J.‐D., and M.C. wrote the manuscript with input from M.E.G.D.A., J.M., L.A.H., A.B.P.J.‐D. acquired funding. All authors have read and agreed to the published version of the manuscript.

## Conflicts of Interest

The authors declare no conflicts of interest.

## Supporting information


Figures S1–S3.

## Data Availability

The sequencing data have been deposited in GEO under accession number GSE290476. The datasets supporting this study are available from the corresponding authors upon reasonable request.
