# Supplementary material for: TFEB Orchestrates Stress Recovery and Paves the Way for Senescence Induction in Human Dermal Fibroblasts
Source: Aging Cell. 2025 May 1;24(7):e70083. doi: 10.1111/acel.70083 (PMC12266763; doi:10.1111/acel.70083)
Supplement: Supplementary file 1 — Figures S1–S3. [file ACEL-24-e70083-s001.docx]

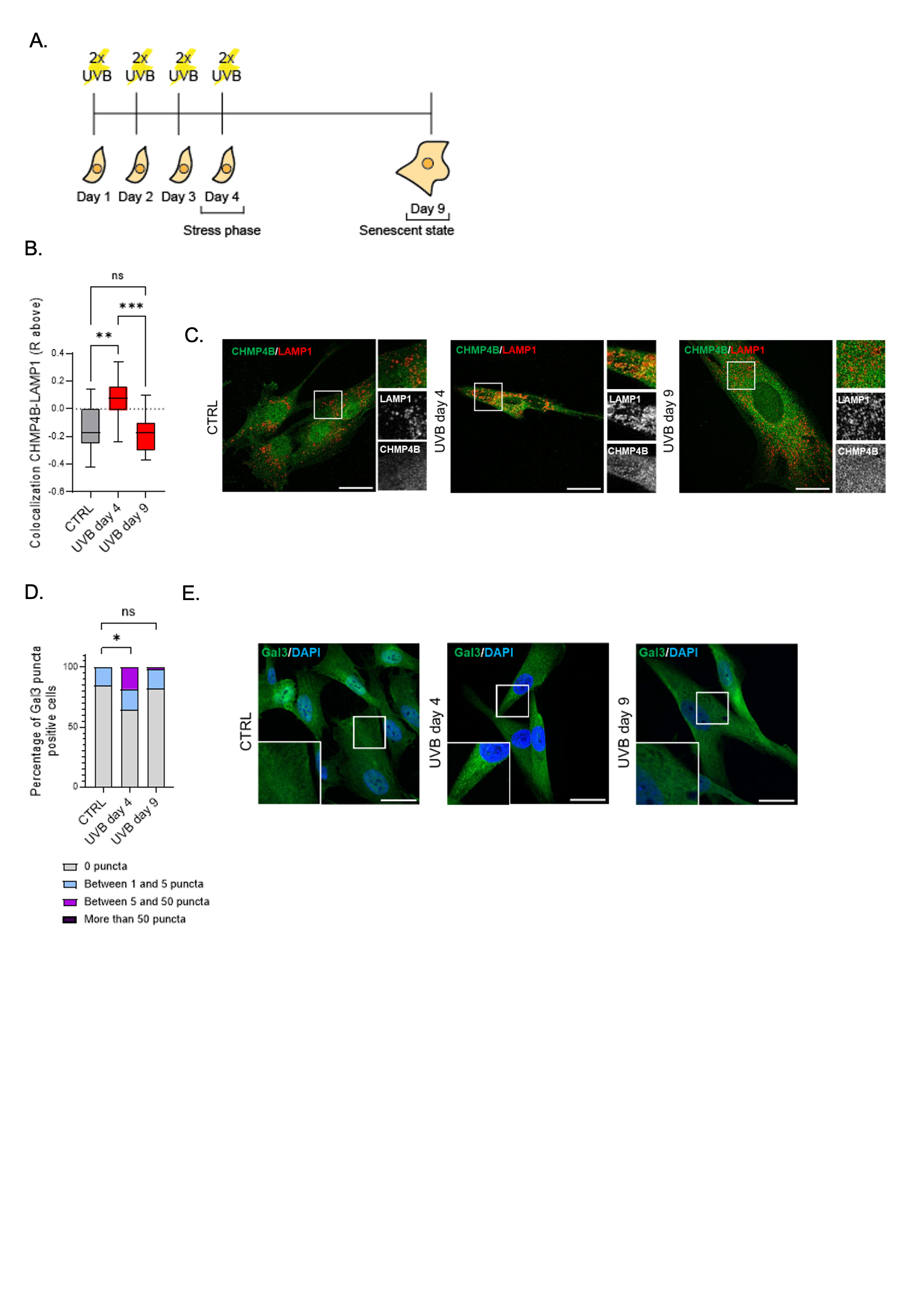


**Supplementary Figure 1. Lysosomal damage is increased by UVB treatment.** A. Scheme showing UVB SIPS induction on HDF. B. Analysis of CHMP4B-LAMP1 colocalization in UVB-stressed (UVB day 4) and UVB-senescent (UVB day 9) HDFs. C. CHMP4B-LAMP1 immunofluorescence representative pictures from control, UVB-stressed (day 4) and UVB-senescent (day 9) HDFs. D. Galectin3 puncta analysis from control, UVB-stressed (day 4) and UVB-senescent (day 9) HDFs. E. Galectin3 immunofluorescence representative pictures from control, UVB-stressed (day 4) and UVB-senescent (day 9) HDFs. Scale bars represent 30µm. Data represents mean values ± SD, N=3. In all graphics ns: non-significant, *p < 0.05, **p < 0.01, ***p < 0.001, ****p < 0.0001.


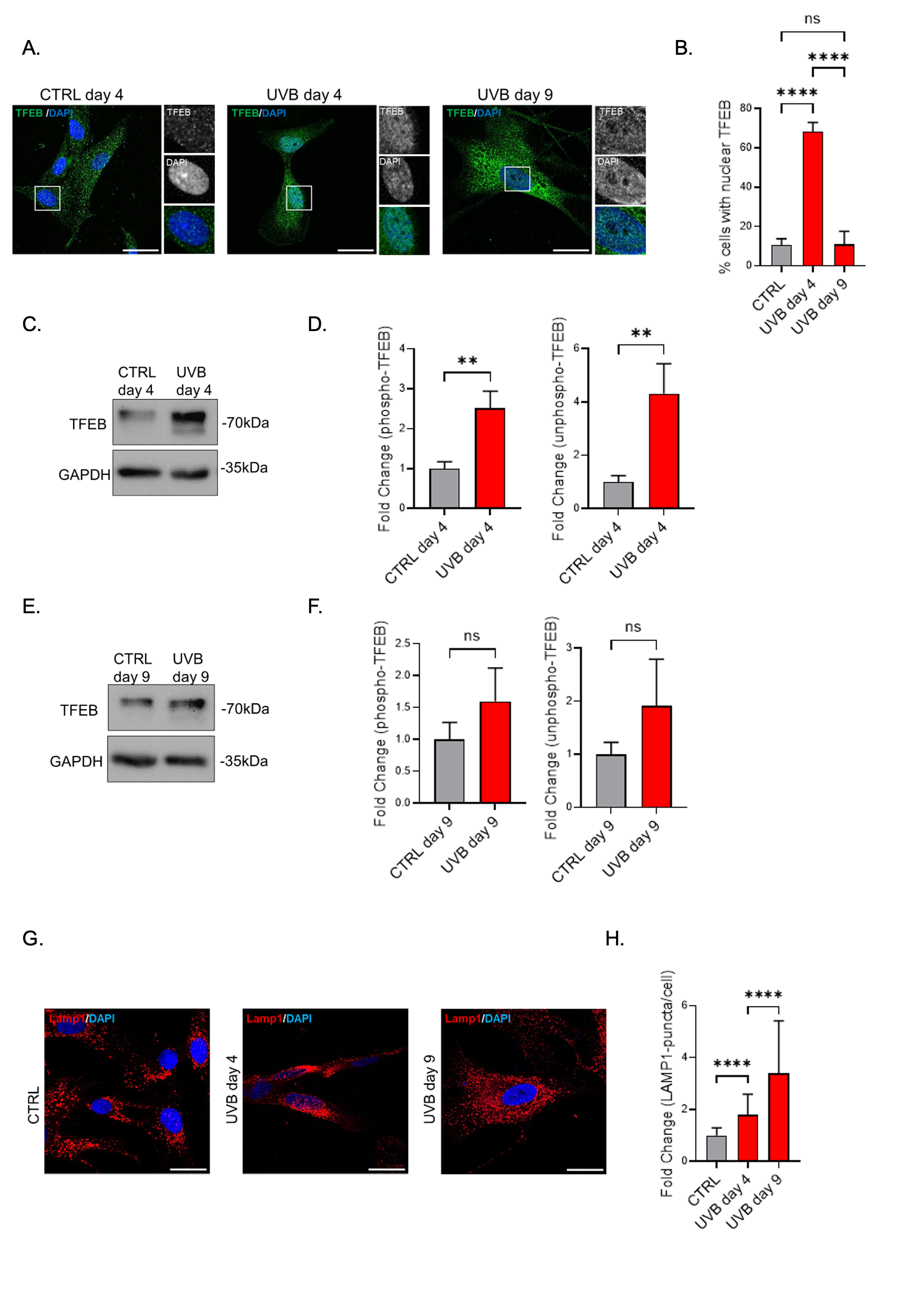


**Supplementary Figure 2. TFEB is active during UVB stress-phase but its activity diminishes during senescent state.** A. TFEB immunofluorescence representative pictures from control, UVB-stressed (day 4) and UVB-senescent (day 9) HDFs. B. Quantification of nuclear TFEB percentages in control, UVB-stressed (day 4) and UVB-senescent (day 9) HDFs. C. TFEB western blot image in UVB-stressed (day 4) HDFs. D. Densitometric analysis of phosphorylated and unphosphorylated TFEB bands in UVB-stressed (day 4) HDFs. E. TFEB western blot image in UVB-senescent (day 9) HDFs. F. Densitometric analysis of phosphorylated and unphosphorylated TFEB bands in UVB-senescent (day 9) HDFs. G. LAMP1 immunofluorescence representative pictures from control, UVB-stressed (day 4) and UVB-senescent (day 9) HDFs. H. Comparative analysis of the fold change in LAMP1 puncta in LAMP1 immunofluorescence pictures among control, UVB-stressed (day 4) and UVB-senescent (day 9) HDFs. Scale bars represent 30µm. Data represents mean values ± SD, N=3. In all graphics ns: non-significant, *p < 0.05, **p < 0.01, ***p < 0.001, ****p < 0.0001.


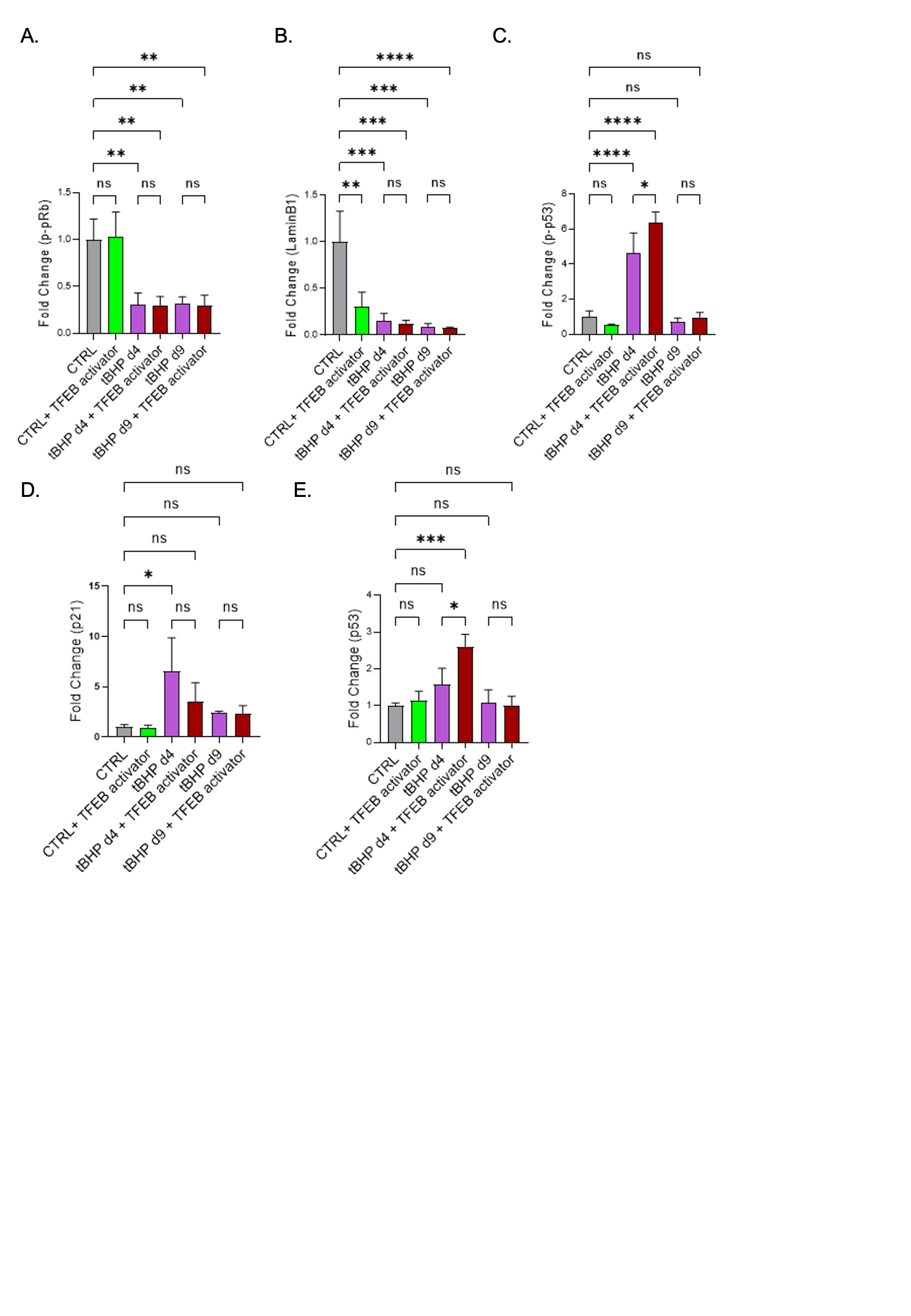


**Supplementary Figure 3.** A-E: Densitometric analysis of senescence markers protein expression in tBHP treated HDFs day 4 and day 9 +/- TFEB activator. Data represents mean values ± SD, N=3. In all graphics ns: non-significant, *p < 0.05, **p < 0.01, ***p < 0.001, ****p < 0.0001.
